# Uniportal thoracoscopic extended right apical segmentectomy with virtual-assisted lung mapping: a case report

**DOI:** 10.1186/s40792-023-01757-x

**Published:** 2023-10-03

**Authors:** Masahiro Yanagiya, Ami Wada, Nobuyasu Awano, Takehiro Izumo, Yoshiaki Furuhata

**Affiliations:** 1 Department of Thoracic Surgery, Japanese Red Cross Medical Center, 4-1-22 Hiroo, Shibuya-Ku, Tokyo, 150-8935 Japan; 2 Department of Respiratory Medicine, Japanese Red Cross Medical Center, Tokyo, Japan

**Keywords:** Thoracic surgery, Lung cancer, Segmentectomy, Uniportal VATS

## Abstract

**Background:**

Pulmonary extended segmentectomy is an optional surgical treatment for early-stage non-small cell lung cancer that helps to achieve optimal surgical margins. Here, we describe a challenging instance of extended segmentectomy via uniportal video-assisted thoracic surgery with virtual-assisted lung mapping, a preoperative bronchoscopic dye marking procedure.

**Case presentation:**

A 72-year-old woman presented with two tumors that were clinically diagnosed as early-stage lung cancer; extended right apical segmentectomy was indicated. Because the tumors had appeared unidentifiable intraoperatively, we performed virtual-assisted lung mapping for tumor localization and delineation of the optimal resection area. Surgery was conducted through a single port. All virtual-assisted lung mapping markings were visible. After dissection of the apical vessels and bronchi, a putative intersegmental line was determined using collateral ventilation. Based on the putative intersegmental plane identified by collateral ventilation and the virtual-assisted lung mapping markings, the resection line was delineated. Extended apical segmentectomy along the resection line was successfully performed via uniportal video-assisted thoracic surgery. The postoperative course was uneventful. The pathological diagnosis was minimally invasive adenocarcinoma and adenocarcinoma in situ.

**Conclusions:**

Virtual-assisted lung mapping can help to achieve optimal extended segmentectomy via uniportal video-assisted thoracic surgery.

**Supplementary Information:**

The online version contains supplementary material available at 10.1186/s40792-023-01757-x.

## Background

Segmentectomy has become an optimal surgical treatment for early-stage non-small cell lung cancer [1–3]. In contrast to lobectomy, segmentectomy can preserve lung parenchyma and thus contribute to favorable outcomes, as previously reported [1–3]. However, segmentectomy can increase the rate of local recurrence compared with lobectomy [1, 4]. The surgical margin is a key factor that influences the achievement of an oncologically successful segmentectomy [4, 5].

Extended segmentectomy is an important approach that can help to achieve optimal resection margins [6]. In extended segmentectomy, the resection line can cross the intersegmental boundary if a portion of the neighboring segment is cut in a non-anatomical manner [5]. Thus, lesion localization is necessary to determine the extent of resection in extended segmentectomy [5].

Virtual-assisted lung mapping (VAL-MAP) is a well-established preoperative bronchoscopic dye marking technique that utilizes virtual bronchoscopic navigation [7, 8]. VAL-MAP provides multiple markings on a map of the pulmonary surface that can be used to localize impalpable tumors and indicate resection lines [7]. VAL-MAP is reportedly effective even in extended segmentectomy because precise localization can help to achieve a sufficient surgical margin [5, 9].

In the past 10 years, uniportal video-assisted thoracoscopic surgery (VATS) has become one of the minimally invasive approaches [10, 11]. Uniportal VATS is associated with less trauma, less pain, shorter hospitalization, and lower complication rates [12–15].

Here, we describe a challenging instance of extended segmentectomy for early-stage non-small cell lung cancer via uniportal VATS. By utilizing VAL-MAP, we achieved sufficient surgical margins and performed optimal extended segmentectomy.

## Case presentation

**Fig. 1:**
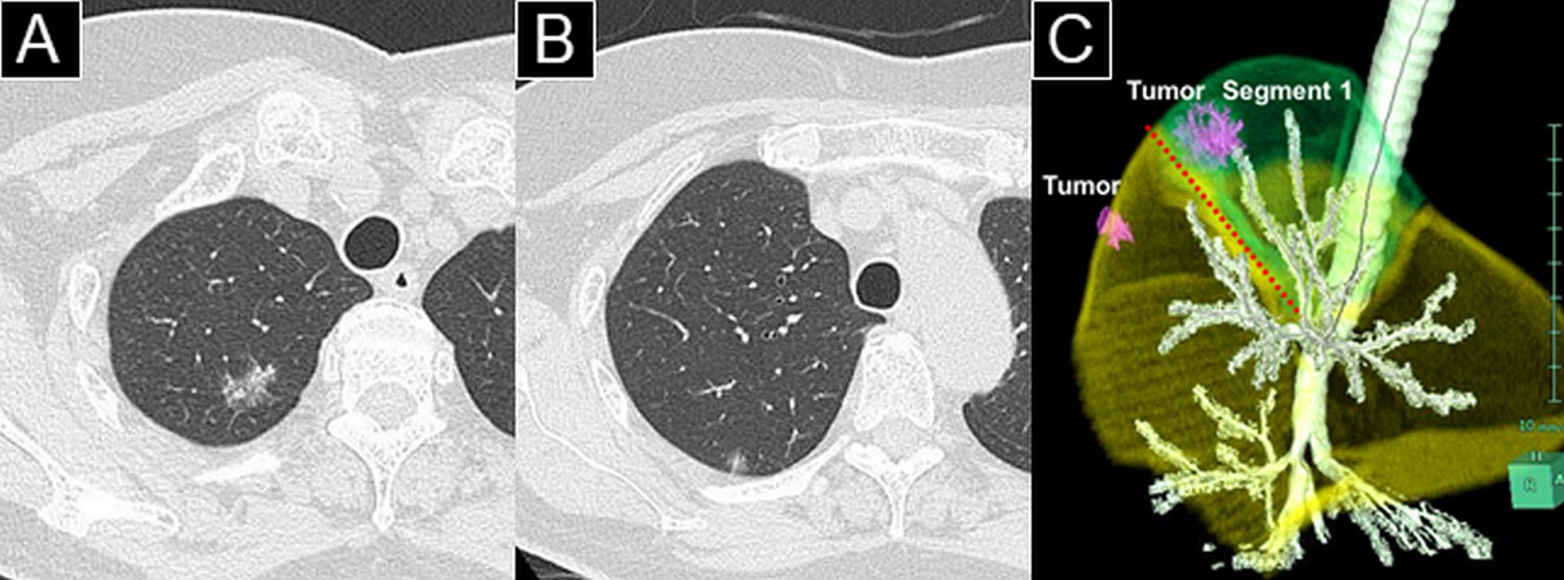
Preoperative chest computed tomography images and a three-dimensional image of the tumors. **A** Chest computed tomography showed a partially solid ground-glass nodule located in right segment 1. **B** A pure ground-glass nodule was located in right segment 2. **C** The tumor in segment 1 (green area) was near the boundary between segments 1 and 2 (red dotted line)

A 72-year-old asymptomatic woman with a medical history of unstable angina pectoris and diabetes mellitus, with an allergy to iodinated contrast media, was referred to our hospital for management of two abnormal ground-glass lung nodules that had been detected by chest computed tomography (CT) during her annual medical examination. During 4 years of close follow-up, one of the nodules had gradually become larger. Chest CT revealed a partially solid ground-glass nodule (overall size, 17 mm; solid component, 5 mm) located in right segment 1 (apical segment) (Fig. 1A, Additional file 1: Video S1); a pure ground-glass nodule (overall size, 7 mm) was located in right segment 2 (posterior segment) (Fig. 1B, Additional file 1: Video S1). 18F-fluorodeoxyglucose positron emission tomography (FDG-PET) detected hypometabolic activity (maximum standardized uptake value, 2.6) in the larger nodule. No distant metastasis was identified by whole-body CT or FDG-PET. Based on the latest TNM classification [16], the nodules were suspected to constitute two early-stage non-small cell lung cancers (cT1miN0M0-IA1 and cTisN0M0-0), both of which were candidates for surgery.

Segmentectomy was indicated for the nodule in segment 1, and wedge resection was indicated for the nodule in segment 2. The nodule in segment 1 was located near the border between segments 1 and 2 (Fig. 1C, Additional file 1: Video S1). Extended right apical segmentectomy was planned to concurrently resect both tumors with sufficient surgical margins. VAL-MAP was also planned to localize both tumors and delineate an appropriate resection line for extended segmentectomy.

One day before surgery, we performed VAL-MAP with the patient under local anesthesia and mild sedation. The procedure was conducted as previously reported [7, 9]. Using X-ray fluoroscopy and virtual bronchoscopic navigation, a metal-tipped catheter (PW-6C-1; Olympus, Tokyo, Japan) was introduced into the pleura and indigo carmine was injected [7, 9]. Four dye markings were created on the pulmonary surface. After the bronchoscopic procedure, a chest CT image was recorded to confirm the locations of the four markings and both tumors [7, 9]. These markings were intentionally created to identify the resection line for extended segmentectomy and to localize the tumors.

**Fig. 2:**
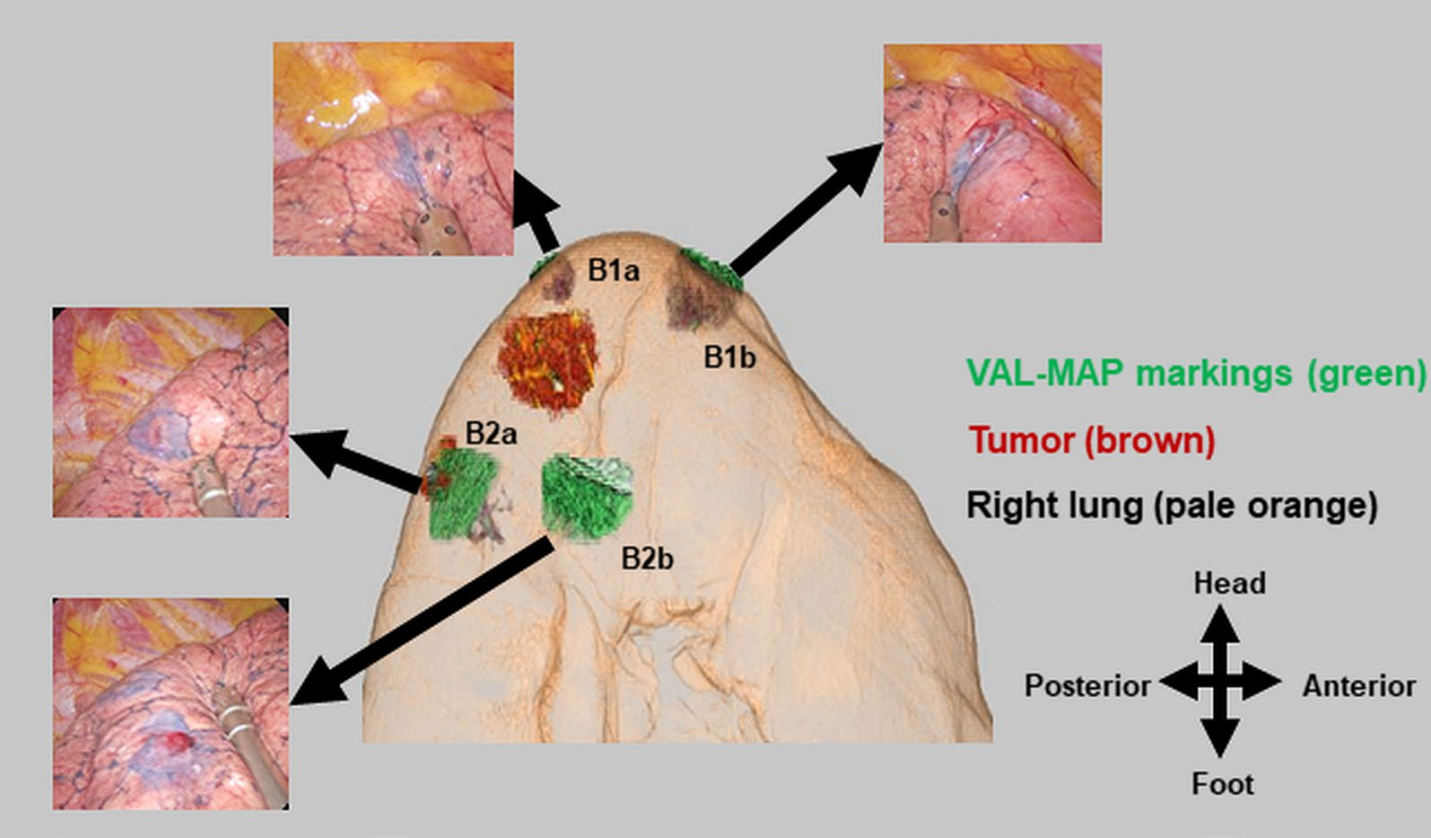
Post-mapping three-dimensional image (center) and photographs (arrows) of the markings.All dye markings were highly visible

Surgery was performed via uniportal VATS with the patient under general anesthesia. A single 4-cm incision was made in the fourth intercostal space without rib spreading. All four VAL-MAP markings were visible (Fig. 2, Additional file 1: Video S1). According to the VAL-MAP marking grading system, two markings in the peripheral branches of B1a and B2a were regarded as grade 2 [7]; the other two markings (in B1b and B2b) were regarded as grade 3 [7] (Fig. 2, Additional file 1: Video S1).

Before the manipulation of anatomical structures, standing stitches using 4–0 Prolene were made for each marking (Additional file 2: Video S2). Initially, the mediastinal pleura were dissected. After confirmation that V1 (apical pulmonary vein) was draining from the right apex, we ligated and dissected V1 (Additional file 2: Video S2). We dissected V1 at the root as reported previously [17]. A3 branched immediately behind V1; it shared a common trunk with A1 and recurrent A2. We exposed and taped A1, then divided it using a mechanical stapler. We identified a prominent lymph node behind A3. The lymph node was sampled and submitted for frozen section diagnosis; no signs of metastasis were detected. The central vein was exposed as far as 1 cm peripheral to the bifurcation of A3a and A3b, which was located 1 cm distal to the bifurcation of A3 and A1. We exposed B1 (i.e., right apical segmental bronchus), then ligated and divided it (Additional file 2: Video S2). We placed additional sutures on the remaining B1 stump. All anatomical branches of vessels and bronchi were divided. Hilar structures were released to create an optimal resection line.

**Fig. 3:**
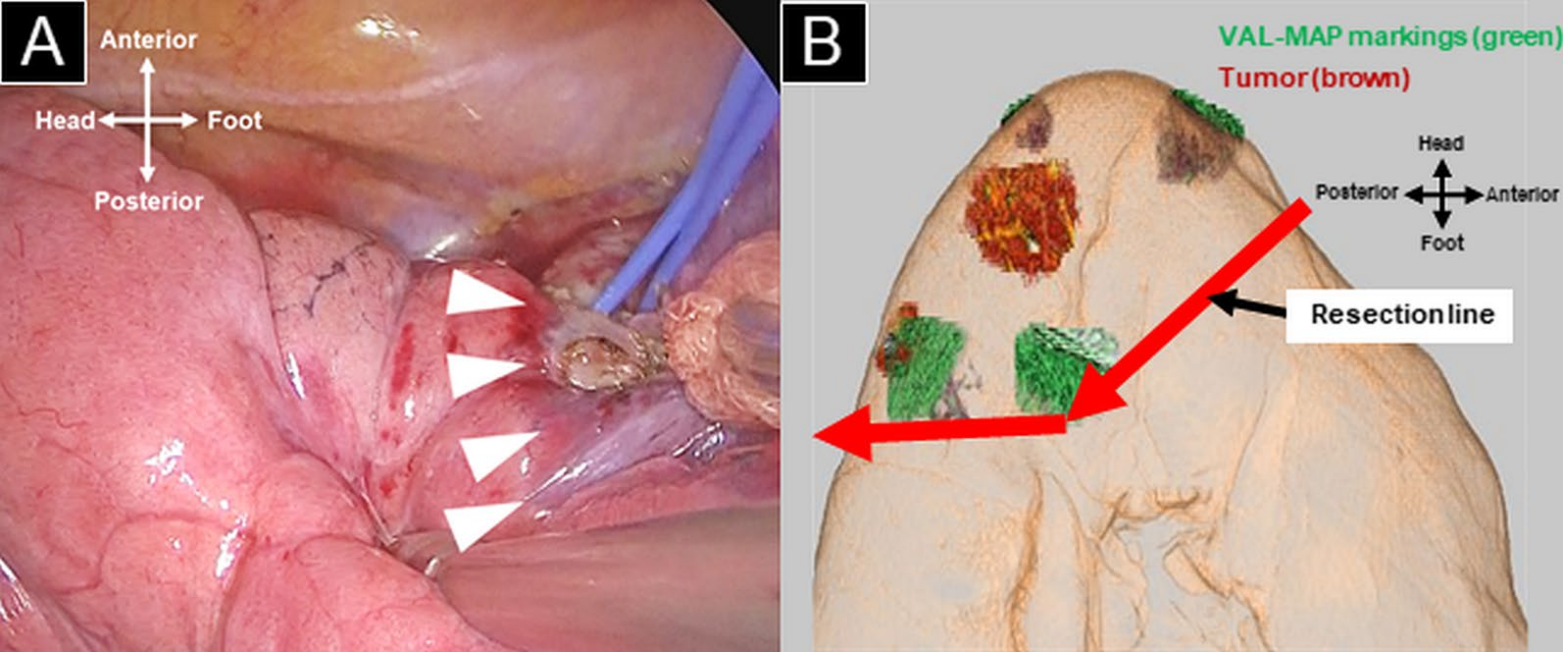
Intersegmental line and resection line.** A** Putative intersegmental line (white arrowheads) between S1 and S3 at hilum, as determined by collateral ventilation. **B** Three-dimensional image showing resection line

**Fig. 4:**
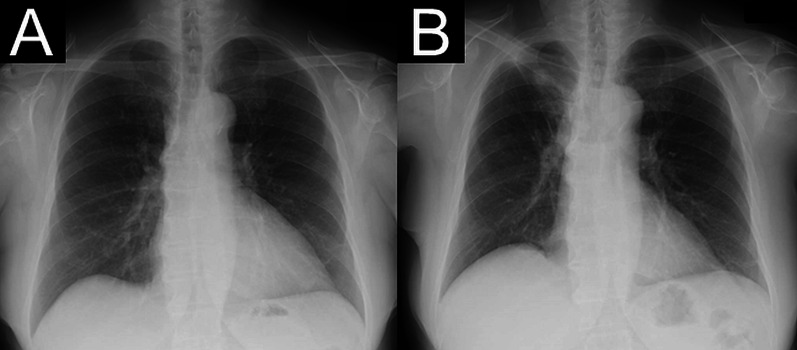
Chest X-ray before and after surgery. **A** Chest X-ray taken before surgery. **B** Chest X-ray taken 1 month after surgery

Intersegmental planes were identified by collateral ventilation, as previously reported [18]. Specifically, the collapsed lung was fully expanded with controlled airway pressure; the target segment (S1) was then fully expanded by collateral ventilation. After 15 min of single-lung ventilation, with the target segment inflated and the other segments collapsed, intersegmental lines were observed [18]. The intersegmental line between S1 and S3 was clearly identifiable, particularly in the hilum (Fig. 3A). Based on the VAL-MAP markings and putative intersegmental lines determined by collateral ventilation, we designed resection lines to achieve extended right apical segmentectomy (Fig. 3B). Using VAL-MAP markings, we readily identified the nodule in segment 1 using a metallic suction tube. However, we were not able to identify the nodule in segment 2. We used mechanical staplers to divide the pulmonary parenchyma along these resection lines and were able to check the surgical margin of the nodule in segment 1 at this time (Fig. 3B). The resected specimens exhibited surgical margins of > 2 cm for both tumors. Subsequently, hilar lymph node sampling was performed. The patient’s postoperative course was uneventful. The chest tube was removed on postoperative day 2, and the patient was discharged without complications on postoperative day 6. The final pathological diagnoses were minimally invasive adenocarcinoma (invasion size, 5 mm; total size, 13 mm) without lymph node metastasis, as well as adenocarcinoma in situ. The chest X-ray at 1 month after surgery showed that the residual right lung had expanded well compared with its size on the preoperative chest X-ray (Fig. 4). At 6 months postoperatively, the patient did not exhibit signs of recurrence.

## Discussion

In this case, we successfully performed uniportal VATS extended right apical segmentectomy with VAL-MAP. Although uniportal VATS segmentectomy is commonly performed, uniportal VATS extended segmentectomy has rarely been reported to our knowledge. Therefore, this case report can provide important insights concerning uniportal VATS extended segmentectomy. Accurate tumor localization, intersegmental plane identification, and optimal resection line creation were key factors in the success of this procedure.

Accurate tumor localization was achieved with VAL-MAP. VAL-MAP will be particularly useful in the management of patients with multiple pulmonary nodules, as demonstrated in the present case [8]. Because VAL-MAP provides multiple markings, multiple nodules can easily be localized [8]. Moreover, VAL-MAP is a safe procedure [8]. A meta-analysis of VAL-MAP revealed that pleural injury and pulmonary hemorrhage occur in approximately 6% and 1% of patients, respectively [8]. A prospective trial of VAL-MAP showed that only a few patients experienced adverse events requiring treatment [7]. Because both tumors contained ground-glass opacities in this case, they were predicted to be impalpable and intraoperatively undetectable as shown in a previous report [19]. Moreover, in our institute, VAL-MAP is indicated for lesions such as those containing ground-glass opacity, those with a tumor diameter equal to or less than 5 mm or those whose distance from the visceral pleura was larger than the tumor diameter as suggested in a previous article [7]. For those reasons, VAL-MAP was indicated for the present case.

In the present case, collateral ventilation was effective in identifying intersegmental planes. A previous report showed that successful identification could be achieved in more than 95% of cases; collateral ventilation is effective, safe, and simple [18]. Although studies in the past 15 years demonstrated the efficacy of systemic intravenous indocyanine green (ICG) injection [20, 21], we avoided the use of ICG because the patient had an allergy to iodinated contrast media. Although we actually dissected V1 at the root as previously reported [17], we should have preserved V1b to identify intersegmental plane. Preservation of V1b running between S1b and S3 is important to achieve anatomically accurate right apical segmentectomy.

The creation of optimal resection lines is a key component of extended segmentectomy. VAL-MAP and collateral ventilation facilitated the delineation of suitable resection lines (Fig. 3). As mentioned above, VAL-MAP is effective in tumor localization; collateral ventilation is effective in identifying intersegmental planes. These two methods complement each other; when used in combination, they can assist extended segmentectomy with the uniportal VATS approach.

Although uniportal VATS is very patient-friendly, it requires considerable surgical skill because all procedures must be performed in a single small port. Uniportal VATS segmentectomy is often challenging because of difficulties in tumor localization and resection line delineation [22]. Using VAL-MAP, which aids tumor localization and helps to identify appropriate resection lines [22], we performed uniportal VATS in a reliable and safe manner. Moreover, we showed that collateral ventilation is feasible, even with the uniportal VATS approach.

## Conclusions

We successfully performed uniportal VATS extended right apical segmentectomy with VAL-MAP. VAL-MAP and collateral ventilation may be useful in achieving optimal extended segmentectomy with the uniportal VATS approach.

### Supplementary Information

**Additional file 1: Video S1.** Images of preoperative, VAL-MAP and intraoperative findings.

**Additional file 2: Video S2.** Surgical procedure.

## Data Availability

All data generated or analyzed are included in this article.

## Abbreviations

CT: Computed tomography
FDG-PET: 18F-fluorodeoxyglucose positron emission tomography
ICG: Indocyanine green
VAL-MAP: Virtual-assisted lung mapping
VATS: Video-assisted thoracoscopic surgery
